# Lungs deposition and pharmacokinetic study of submicron budesonide particles in Wistar rats intended for immediate effect in asthma

**DOI:** 10.17179/excli2016-845

**Published:** 2017-03-10

**Authors:** Abdul Rauf, Aseem Bhatnagar, S.S. Sisodia, Roop K. Khar, Farhan J. Ahmad

**Affiliations:** 1 Formulation Research Laboratory, Department of Pharmaceutics, Faculty of Pharmacy, Jamia Hamdard, New Delhi, India; 2 Department of Nuclear Medicine, Institute of Nuclear Medicine & Allied Sciences (INMAS), Brig. Mazumdar Road, Delhi, India; 3 Bhupal Nobles College of Pharmacy, Udaipur, India

**Keywords:** budesonide, asthma, submicron particles, aerosolization, lungs deposition, pharmacokinetic

## Abstract

The purpose of the present investigation was to study the aerosolization, lungs deposition and pharmacokinetic study of inhalable submicron particles of budesonide in male Wistar rats. Submicron particles were prepared by antisolvent nanoprecipitation method and freeze-dried to obtain free flowing powder. The freeze-drying process yielded dry powder with desirable aerodynamic properties for inhalation therapy. An in-house model inhaler was designed to deliver medicine to lungs, optimized at dose level of 10 mg for 30 sec of fluidization. The *in vitro* aerosolization study demonstrates that submicron particles dissolve faster with improved aerosolization effect as compared to micronized budesonide. Both submicron and micron particles were compared for *in vivo* lungs deposition. The results showed that relatively high quantity of submicron particles reaches deep into the lungs as compared to micron particles. Most pronounced effect observed with submicron particles from pharmacokinetic parameters was the enhancement in peak plasma concentration (C_max_) by 28.85 %, and increase in area under concentration curve (AUC_0-8h_) by 30.33 % compared to micron sized particles. The results suggested that developed submicronized formulation of budesonide can be used for pulmonary drug delivery for high deposition to deep lungs tissues.

## Introduction

Inhaled corticosteroids (ICSs) are effective in chronic inflammation associated with asthma, reported to improve pulmonary function and are recommended as first-line therapy for asthmatic patients (Pornputtapitak et al., 2014[13]). There is considerable evidence that treatment with anti-inflammatory ICSs reduces morbidity and mortality in asthma (Stoloff et al., 2004[17]). ICSs appeared to have a place in the management of severe chronic obstructive pulmonary disease (COPD), perhaps by reducing the frequency of exacerbation and improvement in their quality of life (Chou and D'Urzo, 2014[3]).

In recent years, pulmonary dosage forms have established an important role in localized treatment of lungs diseases. Systemic delivery through the lungs is also emerging as an alternate route due to richly blood perfused surface area, avoidance of first-pass metabolism, and reduces drug degradation that may occur in the gastrointestinal tract (Foster et al., 2001[7]; Rasenack et al., 2003[14]). Pulmonary drug delivery approaches continue to develop rapidly in an effort to improve product stability and efficacy for local and systemic treatment of diseases (Chougule et al., 2007[4]). The main problem with pulmonary drug delivery is the poor deposition efficiency. In some cases only 10 % of the inhaled drug powder reaches the alveoli (Chougule et al., 2007[4]). 

In addition, many current and emerging formulations would benefit from improved drug dissolution rate, which often enhances drug bioavailability. In the past, significant efforts have been dedicated to expand nanotechnology for drug delivery since it offers a potential means of improving the delivery of small molecule drugs as well as macromolecules such as proteins, peptides, or genes to the tissues of interest (Umeyor et al., 2001[18]; Bateman et al., 2008[2]). Increase in the percentage of poorly water-soluble molecules being identified as active pharmaceutical ingredients beckons new approaches to bring these molecules to the market place in a timely fashion (Merisko-Liversidge et al., 2003[12]). Nanoparticle suspension used in nebulizers or metered dose inhalers often suffers from physical instability in the form of uncontrolled agglomeration or Ostwald ripening. In addition, processing of such suspensions into dry powders can yield broad particle size distributions (El-Gendy et al., 2009[6]). There are many advantages for developing dry powder inhalable (DPI) formulations for pulmonary drug delivery, which include reduced dosing frequency, improved patient compliance and reduction of side effects (Roche and Huchon, 2005[15]).

Budesonide (BUD), an inhaled corticosteroid (ICS), is one of the most valuable therapeutic agents for the prophylactic treatment of moderate to severe asthma. Budesonide is reported to have high ratio of topical anti-inflammatory to systemic activity and is one of the most extensively used inhaled corticosteroid (Roche and Huchon, 2005[15]; Daley‐Yates, 2015[5]). Budesonide also decreases airway hyper-responsiveness and reduces the number of inflammatory cells and mediators present in the airways of patients with asthma. ICS reach their site of action and the receptor by diffusing into the cells. Therefore, a fast dissolution of the drug molecule in the lungs is desired. The advantages of submicron drug particles are due to their increased solubility and dissolution as compared to micron particles, therefore improving the inhalable properties. The higher saturation solubility and increased dissolution velocity can be explained by the Kelvin-Ostwald-Freundlich and Noyes-Whitney-Prandtl equations (Ali et al., 2016[1]). Adhesiveness of small particles onto the surface of site of action and increase in bioavailability are also among the advantages of submicron particles.

It is well known from powder technology that fine powders/particles generally possess an increased adhesiveness to surfaces compared to larger one. Also, tendency of the particles to stick on mucosal surface at the absorption site enhances absorption rate (Junyaprasert and Morakul, 2015[10]). A prolonged residence time at the site of absorption would be still beneficial, because microparticles are transported out from the lungs by cilia movement, whereas small particles at nano scale adhere a longer time onto the mucosal surface and in that way the submicron particles can be used to increase the absorption of budesonide (Jacobs and Muller, 2002[9]). Submicronized particles generally contain a very low or no fraction of microparticles, which reduces unwanted deposition of particles in mouth and pharynx, therefore decreases local side effects. On the basis of these known principles, the advantages of submicron drug particles are used to improve drug formulations for pulmonary delivery of budesonide. Submicron drug particles can be obtained by different production methods. If the drug is soluble in an organic solvent, precipitation would be a possible method (Gao et al., 2008[8]). 

For the present study, submicron drug particles were prepared by nanoprecipitation method. The main objective of the study was to investigate the aerosolization effect of developed submicron particle and also to carry out lungs deposition and pharmacokinetic study in male Wistar rats. 

## Materials and Methods

### Materials

Micronized budesonide was a generous gift from Lupin India Ltd. (Pune, India). Leutrol F68 was obtained from BASF Co. (Mumbai, India). Different grades of inhalable lactose, mannitol and sorbitol were procured from DFE pharmaceuticals (Bangalore, India). All other chemicals and reagents were of analytical grade and used as received without further purification.

### Methods

#### Preparation of submicronized DPI formulation

1 % budesonide solution was prepared by dissolving in a mixture of organic solvent [acetone: ethanol (3:1 ratio)] and added to deionized water previously mixed with 0.5 % leutrol F68 dropwise with constant stirring at 1200 rpm for 2.5 hrs. Solvent to antisolvent ration was 0.2. After complete evaporation of organic solvent, submicronized suspension was subjected to high pressure homogenization (600 bar/3 cycles) to obtain desired particle size. Dry powder formulation was obtained by freeze-drying process using a mixture of cryoprotectant (drug: lactose: sorbitol: mannitol: 1: 25: 5: 15). Conventional micronized DPI formulation was prepared simply by blending micronized budesonide with lactose to yield free flowing inhalable powder. 10 mg of dry powder formulation was equivalent to 200 µg of budesonide.

#### Aerosolization study

Aerosolization study was performed in the climate chamber (25 °C/65 % RH) using Andersen cascade impactor (ACI). Formulation equivalent to 200 µg of budesonide was released into the ACI consisting of a throat, a preseparator, 8 frames with non-coated plates, a filter connected to a 3-way valve and a vacuum pump (air flow 60 l/min; flow time 8 sec). The budesonide deposited onto each frame and plate, throat, preseparator and filter was dissolved in 50 ml mobile phase (methanol: water; 90:10 %v/v), and the concentration was analyzed using validated HPLC method. The total recovered mass (RM) and the total fine particle mass (FPM) (≤ 5.8 µm) of emitted budesonide were calculated. The respirable fraction (RF) was calculated using the equation;


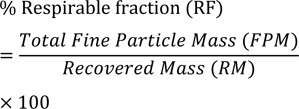


The mass median aerodynamic diameter (MMAD) is the median value of a curve representing the cumulative particle size distribution.

#### Fabrication and working of in-house model inhaler 

**Figure 1:**
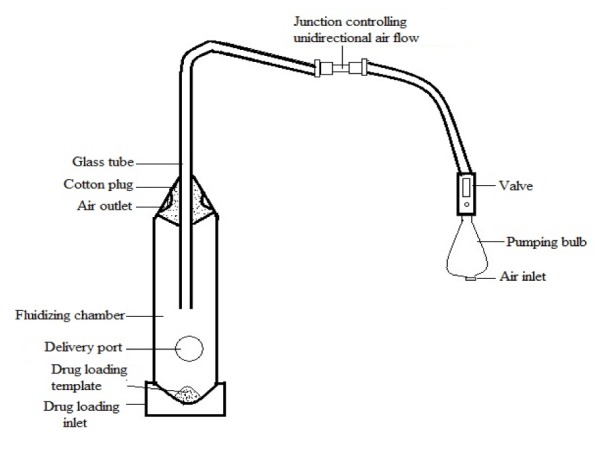
Schematic representation of different parts of in-house model inhaler

In order to deliver submicronized DPI formulation to investigational rodents, an inhalation apparatus (Figure 1(Fig. 1)) was engineered and patented (Jamia Hamdard University; 2012. Indian Patent: 1731/DEL/2010). The conditions were optimized for better delivery by conducting preliminary trials based on loaded amount and fluidization time. In brief, the fluidizing chamber consists of 15 mL glass tube having an even hole about 0.5 cm in diameter on its wall at a distance of about 2.5 cm from the tip. A fine glass tubing of 2 mm internal diameter was inserted into the fluidizing chamber from the top of the taper to clearance of about 2.5-5 cm from the cap's inner surface. It also consists of two holes at the top of fluidizing chamber with 0.1 mm diameter plugged with cotton wool to counter balance the sudden rise in pressure inside the chamber. The glass tubing was connected to a pumping bulb through a plastic tube. 

The plastic tube consists of a junction which controls the unidirectional air flow to prevent drug powder sucked off by vacuum created during relaxation of pumping bulb. The pumping bulb was manually triggered to fluidize the bed of the respective DPI formulation. Complete dispersion of powder bed was achieved with very low volumes of high velocity air coming out of the inlet end of glass tubing to form the desired aerosol. Pre-weighted sample for inhalation were gently put in the central drug loading template placed at cap, which acts as a receptacle and thereafter fastened (screw tight) to the body. 

The sterile cotton wool plug (200 mg in weight) was held over the outer orifice of the fluidizing chamber. The dose fluidized from the in-house inhalation apparatus under comparable conditions of airflow was determined by collecting submicronized and micronized DPI formulation on cotton plug held at delivery outlet when the apparatus was actuated with different amount of sample loaded on the drug loading template. For the system validation, the fabricated apparatus was primed with respective budesonide dry powder formulation by fluidizing five doses and discarded the residue. This procedure led to adsorption of significant amount of DPI on the wall of the apparatus, and increased the amount as well as the reproducibility of dose delivered as compared to unprimed apparatus.

#### Optimization of dose delivery 

After priming of apparatus, the scheme of fluidization was studied between 10-40 sec using different amount of DPI formulations (5-20 mg) to ensure optimum exposure. The rubber bulb of apparatus was actuated with the pressure at the rate of 1 actuation/sec. A sheaf of cotton wool was used to completely occlude the delivery port to observe the quantity available for animal at mouth piece region during exposure. The sheaf surface exposed to fluidized DPI formulations remained flush with the inner wall of the tube and the area of the sheaf exposed to aerosol was quite same in each determination. Finally, the powder laden cotton wool plug was extracted for budesonide in mobile phase and analyzed by validated HPLC method. 

### In vivo lungs deposition and pharmacokinetic study

#### DPI exposure to animals

Authorization to conduct *in vivo* study was obtained from Institutional Animal Ethics Committee, B. N. College of Pharmacy (Reg. No. 870/ac/08/CPCSEA). Male Wistar rats (200-250 g) were provided by the experimental central animal house. The animals were kept under standard laboratory conditions at 25 ± 2 °C temperature and 60 ± 5 % relative humidity, housed in polypropylene cages, with free access to standard laboratory diet (Lipton feed, Mumbai, India) and water ad libitum. A period of 7 days was allowed for acclimatization of rats before any experimental manipulation was undertaken. The rats were fasted overnight before the day of the experiment. After priming of apparatus, the drug loading template of fluidizing chamber was loaded with 10 mg (equivalent to 200 µg budesonide) sample of dry powder and the cap was fastened to the body. Rats were restrained with their snout placed against the peripheral aperture of the apparatus and the pumping bulb was gently actuated to fluidize the sample at the rate of 1 actuation/sec. 

#### Collection of blood samples and tissues

Samples of blood were collected by the method reported in the literature (Shirasaki et al., 2012[16]) with slight modification. Rats were held by grasping the loose skin of the back firmly with fingers and head elevated without spreading the submaxillary gland. Petroleum jelly was applied to fur previously wetted with alcohol, revealing the jugular vein as a blue pulsating area. 27 gauge needles were inserted into the jugular vein through the pectoral muscle below the sternoclavicular junction and 0.5 mL blood was collected at scheduled time point (0.5 min - 8 hrs). Animals were anesthetized by an intraperitoneal injection of xylazine (5 mg/kg) combined with ketamine HCl (50 mg/kg). Tissues removed after dissection was treated with phosphate buffer saline (pH 7.4) to remove unwanted residue. Drug particulates deposited in nasal cavity and on facial hairs were separately collected by washing with 10 mL acetonitrile solution. 

#### Plasma sample preparation 

Collected blood samples were transferred in EDTA-coated microcentrifuge tubes, centrifuged at 10,000 rpm for 10 min. Plasma separated was stored at -21 °C until drug analysis. After thawing plasma samples were mixed with 4 % phosphoric acid solution in 1:1 ratio to release protein-bound drugs (Vieira et al., 2010[19]). The samples were extracted with 3 mL of ethyl acetate. The organic phase was separated by centrifugation at 3,000 rpm for 10 min and evaporated to dryness over a stream of nitrogen gas. The residue was reconstituted with 0.1 mL of mobile phase (methanol: water; 90: 10 %v/v), and analyzed by validated HPLC method. 

Pharmacokinetic parameters were calculated by noncompartmental analysis also called as Model independent analysis using WinNonLin version 4.0 (Pharsight Corp., Mountain View, CA). Maximum plasma concentration (C_max_), time to reach maximum plasma concentration (T_max_), area under the concentration curve from 0-8 hrs (AUC_0-8_), area under the concentration curve for 0 to infinity (AUC_0-∞_), and mean residence time (MRT) were calculated. 

#### Extraction of drug from tissues

Tissues were homogenized in water (1 mL/g) using a Potter S Homogenizer (B. Braun Biotech International, Melsungen, Germany). Tissue homogenates were shaken in a mechanical shaker for 3 min previously mixed with 10 mL acetonitrile and extensively centrifuged at 4000 rpm for 10 min. Supernatant solution was collected separately in a glass tube and liquid-liquid extraction was performed by rolling for 10 min with ether (Lin et al., 2015[11]). After centrifugation the organic layer was transferred into a new tube and evaporated until dry under oxygen free nitrogen (OFN) at 40 °C. The samples were reconstituted with 5 mL mobile phase (methanol: water; 90: 10 %v/v). 10 µL samples were injected and analyzed by the validated HPLC method. Budesonide content was determined by using regression analysis equation. Aliquots obtained by washing of nasal cavity and on facial hairs were also separately collected, extracted and analyzed similarly.

### HPLC condition

The apparatus consisted of Shimadzu (Kyoto, Japan) liquid chromatography equipped with an LC-10ADVP pump and an SPD-10AVP UV detector. Chromatographic separations were performed on a Phenomenex reverse phase C-18 (250 x 4.6 mm, 5 µm) Luna column, coupled with a C_18 _guard column. Data was analyzed using Shimadzu CLASS-VP software (Version 6.2). The budesonide peak was eluted at approximately 4.30 min, at a flow rate of 1 mL/min, injection volume of 10 µL, and absorption wavelength of 240 nm. 

## Results and Discussion

### Aerosolization study

The fine particle dosage (< 5.5 microns) for submicronized DPI formulation, conventional micronized formulation and pure drug were found to be 82.36 %, 60.87 % and 36.72 %, respectively (Table 1(Tab. 1)). The respirable dose (< 3.3 microns) for submicronized formulation was 69.04 %, which was 2.16 times higher than conventional micronized formulation and 4.9 times higher than pure drug. The emitted dose of each formulation was considered as 100 % for *in vitro* deposition study. The mass median aerodynamic diameter (MMAD) were smallest (1.59) compared to other samples, which advocates significant improvement in *in vitro* drug deposition for submicronized formulation.

### Optimization of dose delivery 

The inhalation apparatus assembly was designed to facilitate maximum exposure of fluidized formulation as well as collection of formulation over cotton wool plug in minimal time. The amount of budesonide collected on the cotton wool plugs are presented in Table 2(Tab. 2). The optimal condition to deliver ~200 µg budesonide dose was found to be 30 sec and 40 sec (Rate of actuation: 1 actuation/sec) for submicronized and micronized DPI formulation, respectively. Additionally, this method can be used to examine the behaviour of inhalation dosage form with different aerodynamic particle distribution and the amount of dry powder dose.

### In vivo lungs deposition study 

**Figure 2:**
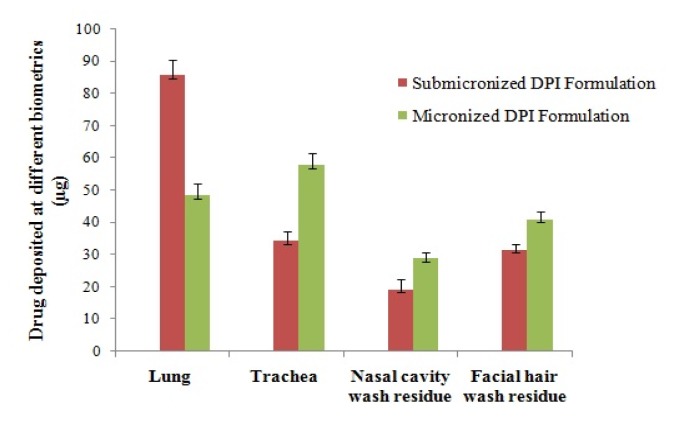
Drug deposited at different biometrics after inhalation to Wistar rats

After inhalation of submicronized and micronized DPI formulation, the content of budesonide deposited at different biometrics of Wistar rats respiratory tract were analyzed (Table 3(Tab. 3), Figure 2(Fig. 2)). Average content deposited on to the lungs, trachea, nasal cavity and facial hairs was 85.70 µg, 34.32 µg, 19.36 µg and 31.70 µg of budesonide, respectively for submicronized DPI formulation. Whereas for micronized DPI formulation, the average content deposited was 48.49 µg, 57.86 µg, 28.83 µg and 41.10 µg, respectively. In general, 85.54 % and 88.14 % were assayed for submicronized and micronized DPI formulation, respectively after actuation of 10 mg dose (equivalent to 200 µg budesonide).

### Pharmacokinetic study

**Figure 3:**
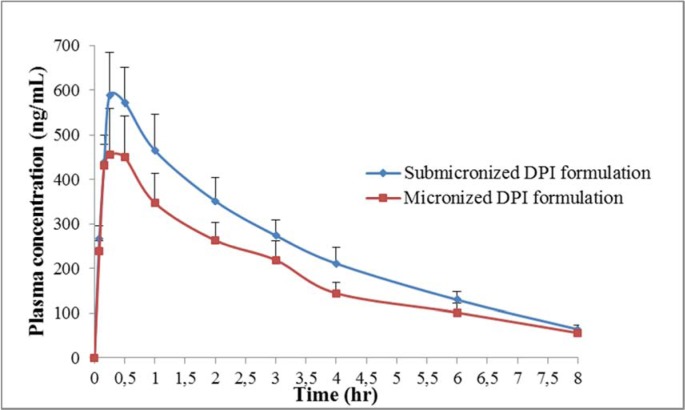
Plasma concentration profile of budesonide after inhalational administration of submicronized and micronized DPI formulation in Wistar rats (n=3). Data represents the mean ± SD (n = 3).

The mean budesonide plasma concentration-time profiles are illustrated in Figure 3(Fig. 3). Estimated pharmacokinetic parameters of budesonide are listed in Table 4(Tab. 4). Observed peak plasma concentration (C_max_) of submicronized and micronized DPI formulation were 588.17 ± 97.5 ng/ml and 456.47 ± 102.4 ng/ml, respectively and the time to reach peak plasma concentration (T_max_) were 0.25 h for both the formulations. C_max_ for submicronized formulation was found to be significantly higher than conventional micronized formulation (*p *< 0.01*)*. The overall area under concentration curve (AUC_0-8h_) for submicronized and micronized formulation were found to be 1989.28 ± 242.8 ng h/ml and 1527.53 ± 222.3 ng h/mL, respectively. Thus, a significantly higher AUC was observed for submicronized DPI formulation compared to micronized formulation (*p *< 0.01*)*. The high value of C_max_ and AUC ensured higher drug availability at the site of action. In general, inhalation exposure resulted in due effect of submicronized DPI formulation by initial burst effect followed by rapid drug released.

The difference in the values of MRT_0-∞_ was not significant (p > 0.05) when the submicronized and micronized formulation was compared, as there is no change in the intrinsic properties of the drug when it was formulated into micronized and submicronized forms.

## Conclusion

The study presents successful development of submicronized DPI formulation of budesonide and their application as a drug carrier in pulmonary delivery to the lungs. Results of *in vitro* aerosolization study depicted better effect of submicron particles compared to micronized particles. *In vivo* lungs deposition study advocated that the submicronized formulation has high and deep lungs deposition of budesonide. The high value of C_max_ and AUC ensured higher drug availability at the site of action. Therefore, the formulation holds a great potential for treating diseases that require direct lungs delivery with reduced dosage and dosing frequency, leading to fewer systemic side effects and improved patient compliance.

## Conflict of interest

All authors have declared there are no conflicts of interest.

## Figures and Tables

**Table 1:**
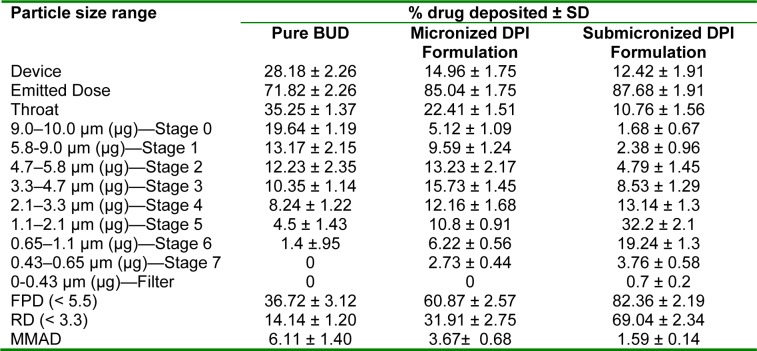
* In vitro *lungs deposition of micronized and submicronized DPI formulation using Andersen cascade impactor (ACI)

**Table 2:**
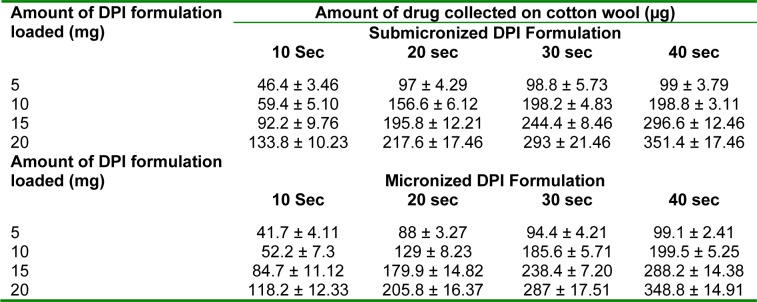
Optimization of dose delivered from in-house model inhaler

**Table 3:**
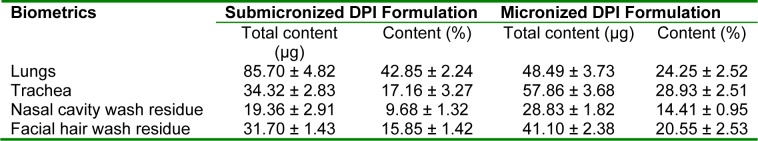
Content deposited at different biometrics after inhalation to Wistar rats (n=3).

**Table 4:**
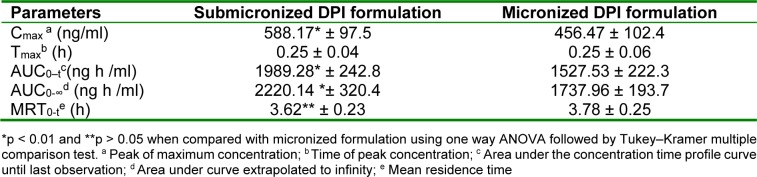
Pharmacokinetic profile of budesonide in Wistar rats (n=3) after inhalational administration
